# Recombination-assisted megaprimer (RAM) cloning

**DOI:** 10.1016/j.mex.2014.05.001

**Published:** 2014-05-20

**Authors:** Jacques Mathieu, Emilia Alvarez, Pedro J.J. Alvarez

**Affiliations:** Department of Civil and Environmental Engineering, Rice University, Houston, TX, United States

**Keywords:** Megaprimer, Restriction-free cloning, PCR cloning, Exponential amplification, Homologous end-joining

## Abstract

No molecular cloning technique is considered universally reliable, and many suffer from being too laborious, complex, or expensive. Restriction-free cloning is among the simplest, most rapid, and cost-effective methods, but does not always provide successful results. We modified this method to enhance its success rate through the use of exponential amplification coupled with homologous end-joining. This new method, recombination-assisted megaprimer (RAM) cloning, significantly extends the application of restriction-free cloning, and allows efficient vector construction with much less time and effort when restriction-free cloning fails to provide satisfactory results. The following modifications were made to the protocol:

• Limited number of PCR cycles for both megaprimer synthesis and the cloning reaction to reduce error propagation.
• Elimination of phosphorylation and ligation steps previously reported for cloning methods that used exponential amplification, through the inclusion of a reverse primer in the cloning reaction with a 20 base pair region of homology to the forward primer.
• The inclusion of 1 M betaine to enhance both reaction specificity and yield.

## Graphical abstract

**Figure:**
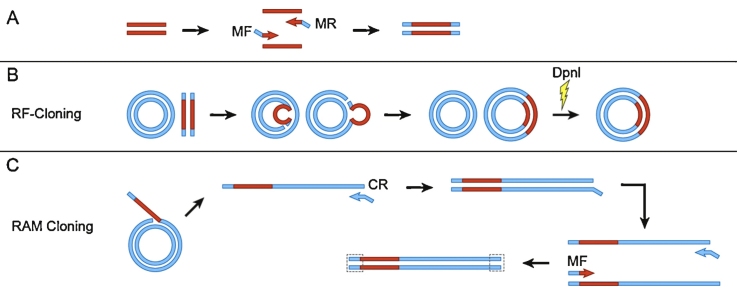

## Method details

RAM cloning is designed to work as either a stand-alone method, or as a means to salvage a failed restriction-free (RF)-cloning attempt. Both RAM and RF-cloning involve the following steps: (i) the synthesis of a megaprimer; (ii) cloning of the megaprimer into the target vector; (iii) removal of parental plasmid from the reaction; (iv) transformation of target organism; and (v) verification of proper insertion. RAM cloning differs from RF-cloning in that it incorporates and additional primer to turn the linear amplification step into an exponential amplification. Additionally, RAM cloning utilizes homologous recombination for end-joining.

### Step 1: megaprimer synthesis

**Fig. 1:**
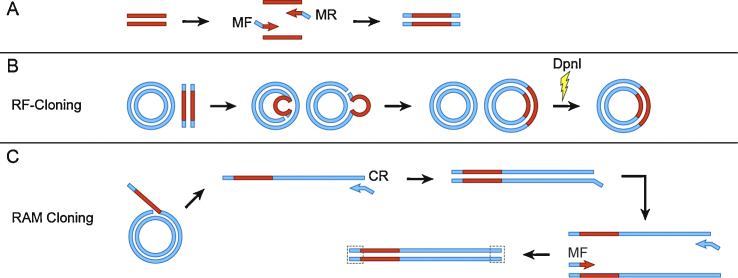
Schematic diagram of RF and RAM cloning. (A) Megaprimers are synthesized by standard PCR with primers harboring 5′ arms homologous to the insertion site on the target plasmid. (B) RF cloning is performed as a modified PCR reaction. The forward and reverse primers are replaced by the megaprimer, which results in linear amplification. The parental plasmid is digested with DpnI prior to transformation. (C) RAM cloning differs from RF cloning by the addition of two primers to the reaction, which enables exponential amplification. The forward primer is the same primer used during megaprimer synthesis. The reverse primer possesses regions of homology to both the target plasmid and the 5′ end of the megaprimer (also homologous to the vector), which facilitates homologous recombination for end-joining subsequent to transformation.

(1) If RAM cloning is being performed subsequent to an attempt to perform RF-cloning, the same megaprimer can be used that was produced for the original RF-cloning, and this step can be bypassed. Otherwise, design primers for PCR amplification of the desired DNA insert following standard primer design methods, but do not exceed 40 base pairs in length (for purely cost-related reasons). Determine the insertion point in the target vector for the DNA insert. Amend the forward primer (MF) with the 20 base pairs of sequence immediately upstream of the insertion site. Amend the reverse primer (MR) with the 20 base pairs of sequence immediately downstream of the insertion site, remembering to use the reverse complement.
(2) PCR amplify the DNA insert using a high fidelity polymerase and the minimum number of cycles necessary to visualize the product (Fig. 1A). Typically we use Kapa HiFi HotStart ReadyMix PCR Kit (Kapa Biosystems) for 15–20 cycles. Reactions consist of 12.5 μL 2X PCR ReadyMix, 0.4 μM each of the MF and MR primers, 10–50 ng DNA template, and H_2_O to 25 μl. Cycling conditions include an initial denaturation step (35 s, 98 °C), followed by 15–20 cycles consisting of a denaturation step (35 s, 95 °C), an annealing step (35 s, average megaprimer *T*_m_), and an extension step (30 s/kbp, 72 °C). This was followed by a final extension step at 72 °C for 5 min.
(3) Verify the megaprimer size, and purify it by gel electrophoresis using a 1% agarose gel. Add 5 μL of 6X loading dye directly to the PCR reaction. Run until sufficient separation has occurred and then gel extract the appropriate band.
(4) Determine megaprimer concentration by UV absorbance.

Alternatively, the optimal number of cycles can be determined by performing real time PCR and the product isolated by stopping the reaction in mid-log phase and performing a standard purification. Product purity can be gauged by examining the strand disassociation curve. However, gel electrophoresis has the advantage of allowing one to separate multiple products.

### Step 2: cloning and transformation

In contrast to RF-cloning which only utilizes the megaprimer and destination vector (Fig. 1B), RAM cloning is conducted *via* a secondary PCR reaction that utilizes the megaprimer, the MF primer, and a second reverse primer (CR) (Fig. 1C). During the initial PCR cycles, the megaprimer disassociates and the strand containing the homologous region at the 3′ end binds to the target vector where it serves as a primer for a linear amplification reaction. One of the resultant linear DNA molecules then serves as a template for the MF and CR primers in a standard PCR reaction. Perform the reaction as follows:

(1) Design the CR primer to possess a 5′, 20-bp overlap with the 5′ region of the MF primer. This overlap is complementary to the region directly upstream of the desired gene insertion site. The rest of the CR primer consists of the necessary sequence upstream of the 20-bp overlapping region on the target plasmid.
(2) Perform standard PCR using a high fidelity polymerase to fuse the megaprimer and target plasmid. A typical reaction might consist of 12.5 μL of Kapa HiFi HotStart ReadyMix PCR Kit (Kapa BioSystems), 0.4 μM each of the MF and CR primers, 10 ng of the target plasmid, a 20:1 molar excess of megaprimer relative to the target plasmid, 1 M betaine, and sterile, nuclease-free water to 25 μL. Set PCR cycling conditions to: an initial denaturation step (35 s, 98 °C), followed by 5–15 cycles of denaturation (35 s, 95 °C), annealing (35 s, 45 °C), and an extension step (30 s/kbp, 72 °C). Follow this with a final extension step at 72 °C for 5 min.
(3) Restriction digest the parental target plasmid DNA. Add 2 μL of DpnI (20 U) directly to the PCR reaction. Incubate at 37 °C for 2 h, and inactivate at 80 °C for 20 min.
(4) Transform the linear plasmid into *E. coli*. A typical protocol may be as follows. Use 3 μL of digested PCR reaction to transform 50 μL of chemically competent *E. coli* cloning-grade cells. Incubate on ice for 10–15 min, heat shock at 42 °C for 30 s, and place back on ice for 1 min. Add 750 μL of recovery media and incubate cells at 37 °C with shaking for 1 h. Centrifuge at max speed for 30 s, pipette off 150 μL of supernatant (do not dispense) and decant remainder in tube. Resuspend cells with the 150 μL of supernatant in pipette and plate on 1% agar plate(s) containing the appropriate antibiotic. Incubate overnight at 37 °C.

### Step 3: cloning assessment

Utilize colony PCR and sequencing to verify the appropriate insert is present and to determine sequence accuracy. Suggested steps include:

(1) Set up 4–6 PCR reactions. A typical reaction might consist of 12.5 μL of Kapa HiFi HotStart ReadyMix PCR Kit (Kapa BioSystems), 0.4 μM each of the forward and reverse sequencing primers, and sterile, nuclease-free H_2_O to 25 μL.
(2) Pick individual colonies with a sterile toothpick or pipette tip. Swab the inside of one of the PCR reaction tubes with the tip containing the colony. Use the same tip to then streak a 1% agar plate containing the appropriate antibiotic. Perform this step for the desired number of colonies and then incubate the plate overnight at 37 °C.
(3) PCR amplify the desired insert region using the following conditions: an initial denaturation step (35 s, 98 °C), followed by 30 cycles consisting of a denaturation step (35 s, 95 °C), an annealing step (35 s, average primer *T*_m_), and an extension step (30 s/kbp, 72 °C). Follow this with a final extension step at 72 °C for 5 min.
(4) Verify the insert size, and purify it by gel electrophoresis using a 1% agarose gel. Add 5 μL of 6X loading dye directly to the PCR reaction and use the entire volume. Run until sufficient separation has occurred and then gel extract the appropriate band. Assess DNA concentration using UV spectroscopy and have the product verified using Sanger sequencing.

**Fig. 2:**
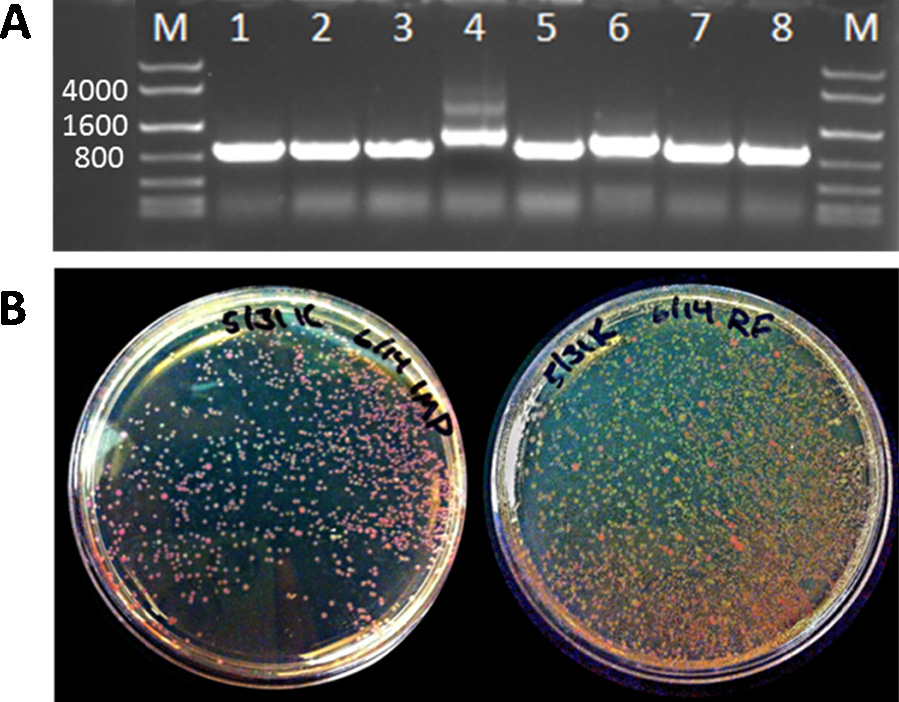
Demonstration of RAM cloning efficacy. (A) Agarose gel electrophoresis of an 847 bp P_tet_-mRFP1 fragment constructed using RAM cloning and amplified by colony PCR from transformed *E. cloni*. Lanes 4 and 6 possessed 5′ repeats that increased the product size. (B) Transformations using RAM cloning (left) or RF cloning reactions (right). Red colonies are properly expressing mRFP1 while beige colonies are considered to harbor incorrect insertions. M: DNA ladder (KAPA Express). (For interpretation of the references to color in this figure legend, the reader is referred to the web version of the article.)

We utilized RAM cloning to insert seven DNA fragments into expression vectors for which we previously had failed during numerous attempts with RF cloning as well as other cloning methods. Results from colony PCR and sequencing indicate that the DNA fragments were correctly inserted in approximately 75% of the colonies examined (Fig. 2 and Table 1). Analysis by gel electrophoresis and sequencing revealed that most of the incorrect colonies possessed repeated sequence at the 5′ end of the insertion site. Earlier experiments suggested that the extent of overlap between the MF and CR primers is a critical factor determining the frequency of obtaining repeated sequence. By limiting overlap to 20 bp, and ensuring another 20 bp of non-overlapping sequence for each primer, we greatly reduced the frequency of repeats.

We compared the efficiency of RF cloning to RAM cloning by inserting a gene encoding mRFP1 under control of the Ptet promoter into a mammalian expression vector (pMEV). In the absence of TetR, this promoter constitutively expresses its associated gene, so we were able to gauge proper insertion by monitoring the production of mRFP1. After cloning and DpnI digestion, the products were purified and the concentrations normalized prior to transformation. While we observed a rather low 16% success rate with RF cloning, the use of RAM cloning increased our success rate to 94% (Fig. 2 and Table 1).

**Fig. 3:**
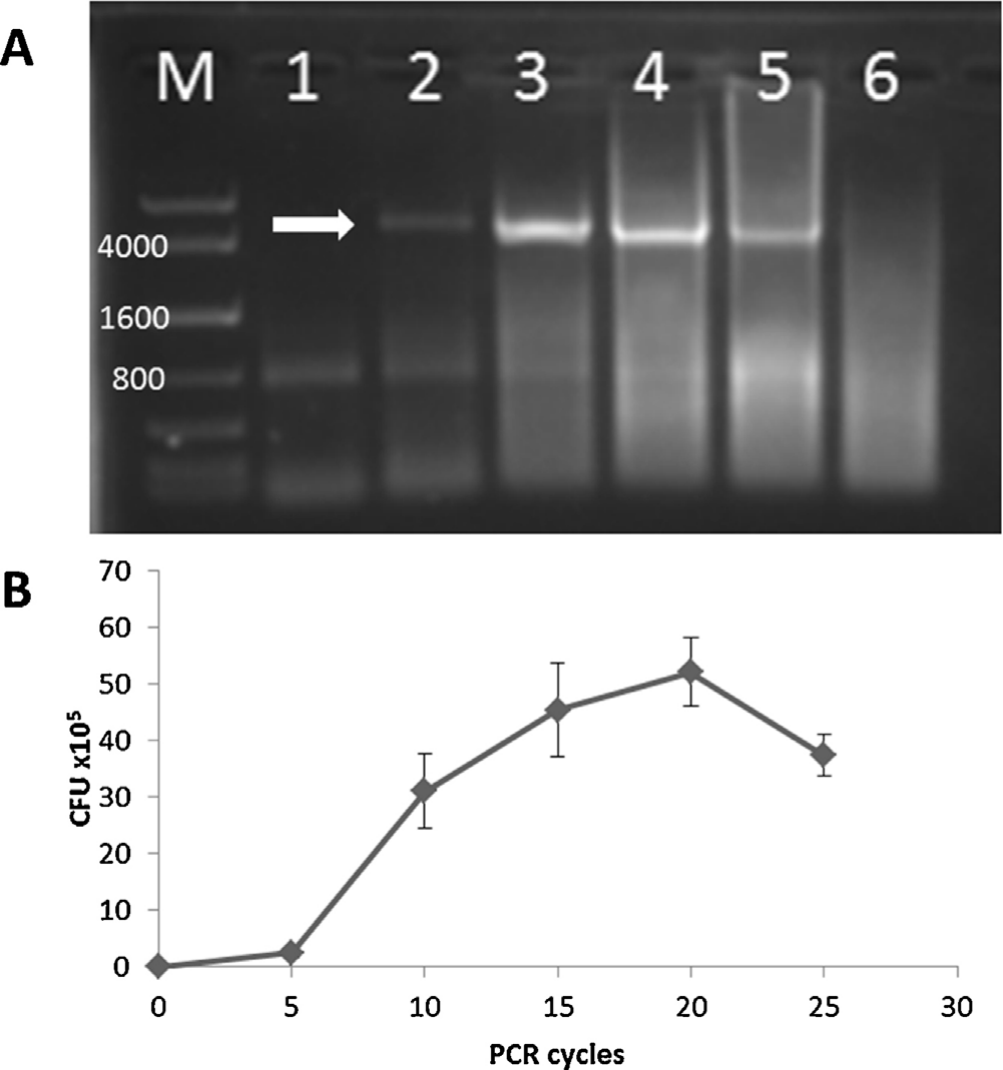
RAM cloning cycle number and cloning efficiency. (A) RAM cloning was performed for insertion of P_tet_-mRFP1 into pMEV using between 5 and 30 cycles (L1: 5, L2: 10, L3: 15, L4: 20, L5: 25, L6: 30). The expected size of the linearized plasmid DNA is approximately 4838 bp (arrow). (B) CFU/μg MP achieved for various cycle numbers. Maximum efficiency was observed using 20 cycles, however sufficient numbers of colonies are obtained using between 5 and 10 cycles. M: DNA ladder (KAPA Express).

A potential limitation with all PCR-based cloning methods is their susceptibility to mutation during product amplification. In order to reduce the introduction of mutations, we utilized only high fidelity polymerase and attempted to limit the number of PCR cycles. During megaprimer synthesis we found that 20 cycles was typically sufficient. We also evaluated the impact of cycle number on cloning efficiency to determine the lowest number of cycles we could utilize while still obtaining constructs containing the proper insert (Fig. 3). While the highest efficiency was observed when utilizing 20 cycles during the RAM cloning reaction, colonies were still obtained when only using 5 cycles. However, to consistently obtain an appropriate number of colonies, we found that 10 cycles was more reliable. Therefore, 30 or fewer total PCR cycles were required to achieve proper DNA fragment insertion in all of the constructs in this study with no mutations detected during sequencing.

For laboratories performing low-throughput cloning of single gene insertions, RF cloning offers a simple, cheap, and mostly effective method for rapid DNA manipulations. However, when RF cloning fails, researchers are often required to utilize completely different cloning methods that require additional design and reagents, and that are often time-consuming. RAM cloning saves time and resources by allowing researchers to utilize the same megaprimer which was produced for RF cloning, and requires only one new primer. Furthermore we found RAM cloning to be more reliable than any other cloning method we have utilized. Our experience suggests that it is not hindered by insert sizes up to at least 2460 bp (maximum size assessed) or GC content up to at least 72% (Table 1). Furthermore, while RF cloning may be less expensive per reaction based on reagent costs, we found that in many cases it would have been more economical to utilize RAM cloning over RF cloning due to the increased amount of screening we have had to perform for some cloning reactions.

## Additional information

### Background

Since the discovery of restriction enzyme-based methods for plasmid construction [1,2], researchers have sought to improve upon the efficiency, reliability, and cost of molecular cloning. Restriction digests can be slow, reduce efficiency, and the need to include (or remove) specific restriction sites in both the insert and vector DNA makes design laborious. Additionally, restriction cloning typically necessitates the introduction of unwanted DNA sequence. Methods such as ligation-independent cloning (LIC) and TA cloning eliminate the need for restriction enzymes, but are dependent on DNA end modifications that are not easily detected, and still suffer from the unintentional introduction of unwanted sequences [3,4]. Other methods, such as USER cloning [5], Gateway cloning [6], In-Fusion cloning [7], Golden Gate cloning [8], and Gibson assembly all have significant advantages, but are often relatively expensive, complex, or unreliable.

Arguably, the most simple and cost efficient method for routine cloning may be restriction-free (RF) cloning. Perhaps attesting to this is that almost identical RF cloning methods have been independently developed by numerous research groups [9–12], and the technique is very similar to another widely used method, circular polymerase extension cloning (CPEC) [13]. In RF cloning, the insert is PCR-amplified using primers that possess 5′ ends that are homologous to the vector. The insert is then used as a megaprimer in a linear amplification reaction with a high-fidelity, non-strand-displacing polymerase. After several PCR cycles, DpnI is used to digest the parental plasmid, and the remaining newly synthesized plasmid (possessing two nicks) is used for transformation.

While fast, inexpensive, and simple to implement, RF cloning does have several drawbacks. Due to its reliance on a linear amplification reaction, product yield is generally low, which hinders downstream monitoring of the cloning reaction and reduces efficiency. Second, RF cloning efficiency further decreases with insert size [10,11]. Additionally, single gene integration events are not always successful, with reliability averaging between 50 and 100%. In our own lab, we frequently use RF cloning and have successfully achieved single gene plasmid integration approximately 80% of the time. Reaction optimization increases the success rate, but for some genes optimization provides no additional benefit, requiring other cloning methods. This not only increases the time and expense, but we have also found that genes which are not amenable to RF cloning, are typically resistant to other cloning methods as well. This could be due to the presence of GC-rich regions or secondary structure near the homologous ends. In these cases, it would be advantageous to monitor reaction progress using agarose gel electrophoresis; however, the linear amplification of RF cloning makes this difficult. Exponential Megapriming PCR (EMP) cloning is a recently reported technique that overcomes these drawbacks of RF cloning by using an exponential amplification step [14]; however, it requires the use of sequential phosphorylation and ligation steps for vector circularization. This significantly increases the time and cost involved in comparison to RF cloning.

In order to address problems associated with molecular cloning failures, we devised a method that could be utilized complementary to RF cloning. The simplicity and convenience of RF cloning makes it an attractive technique for many labs, but the need to troubleshoot and redesign experiments is laborious when it fails. Herein, we present a method to minimize RF cloning failures through a modified reaction that requires only one additional primer. Like EMP cloning, this technique relies on exponential amplification; however, it removes the need for additional enzymatic processing by harnessing *in vivo* homologous recombination for end joining.

## Figures and Tables

**Table 1:** Comparison of successful DNA insertions for RF and RAM cloning.

			Number correct	
ID	Length	GC%	RF	RAM
R5P3E	663	70	0/12	3/4
TA	672	67	4/8	–
R5PI	684	71	0/8	2/4
TPI	753	68	3/8	–
ALDO	918	68	4/4	–
F16BP	969	70	4/4	–
PGI	1248	70	4/4	–
LDS	1251	61	0/16	4/4
G6PD	1485	55	4/4	–
PGM	1575	71	0/8	3/4
TK	1956	70	2/4	–
SP	2460	72	0/10	4/4
Ptet-mRFP1	758	49	58/358	294/312
